# Female adolescent sexual and nonsexual violent offenders: a comparison of the prevalence and impact of risk and protective factors for general recidivism

**DOI:** 10.1186/s12888-015-0615-6

**Published:** 2015-10-07

**Authors:** Claudia E. van der Put

**Affiliations:** Research Institute of Child Development and Education, University of Amsterdam, P.O. Box 94208, 1090 GE Amsterdam, The Netherlands

**Keywords:** Female adolescent sexual offenders, Risk factors, Protective factors, Dynamic factors, General recidivism, Impact on recidivism

## Abstract

**Background:**

This study adds to the scarce literature on female adolescent sexual offenders by examining differences between female adolescent sexual and nonsexual violent offenders in the prevalence and impact of dynamic risk and protective factors for general recidivism.

**Method:**

The sample consisted of female adolescents who were convicted for a sexual offense (FSOs; *n* =31) or nonsexual violent offense (FNSOs; *n* = 407), and for whom the Washington State Juvenile Court Assessment was completed.

**Results:**

In FSOs, considerably more protective and fewer risk factors were present than in FNSOs in almost all domains (i.e., school, relationships, family, attitude and aggression). In addition, differences in the impact of risk/protective factors on general recidivism were found. In FSOs, risk/protective factors in the family and aggression domains were especially important, whereas in FNSOs, risk/protective factors in the attitude domain were especially important.

**Conclusions:**

The results of this study indicate that treatment programs developed for mainstream female offenders may also be useful for female sexual offenders in reducing general recidivism. Furthermore, the results are of importance for determining the main focus of treatment for both mainstream and sexual female adolescent offenders.

## Background

Sexual offending by female adolescents is an understudied area. There are no theories or models specific to female adolescent sexual offending that may be used to guide the treatment process. Little is known about the level of sexual or general recidivism, and even less about the factors linked to recidivism among female adolescent sexual offenders. There are two perspectives explaining juvenile sexual offending. The first perspective focuses on o*ffense-specific risk factors*, such as atypical sexual interests, which are thought to be uniquely, or primarily, relevant to sexual crimes (e.g., [1–3]), whereas the second perspective, the *general delinquency* explanation, assumes that sexual offending is part of a broader pattern of delinquency and could be explained as a manifestation of general antisocial tendencies [4]. This is supported by the fact that the majority of sexual offenders also commit nonsexual offenses (e.g., [5]) and are much more likely to recidivate with a nonsexual crime than a sexual crime (e.g., [3, 6, 7]). In female sexual offenders, general recidivism is also higher than sexual recidivism. A recent meta-analysis of 10 studies on recidivism rates of female *adult* sexual offenders showed that female adult sexual offenders are 10 times more likely to be reconvicted for a nonsexual crime than a sexual crime (~20 % vs. ~2 %; [6]). Therefore, treatment aimed at preventing general instead of sexual recidivism may be more effective for female sexual offenders. However, the dynamic risk/protective factors associated with both sexual and general recidivism among both adult and adolescent female sexual offenders are still unknown [8]. The aim of the present study was therefore to examine the strength of the relations between dynamic risk/protective factors and general recidivism in both female adolescent sexual and nonsexual offenders.

To be able to provide adequate treatment to female adolescent sexual offenders it is necessary to know which dynamic risk and protective factors are related to offending behavior in these girls. Risk and protective factors for offending behavior consists of individual characteristics on the one hand and social characteristics found in the domains of family, peers, school and neighborhood on the other hand [9–11]. These risk factors have been conceptualized as static or dynamic. Static risk/protective factors are circumstances or conditions in a youth’s life, such as intelligence and previously committed offenses, that cannot be changed, whereas dynamic risk/protective factors can potentially be changed, and when changed, result in a corresponding increase or decrease in the risk of offending behavior [12]. Examples of dynamic risk factors are impulsive behavior, positive attitude towards delinquency, anti-social friends, poor parental supervision and truancy and examples of dynamic protective factors are good social skills, good academic performance and support from parents [13].

Dynamic risk/protective factors are often targeted in offender intervention programs and therefore it is important to know which of these factors are strongly related to recidivism [12]. The effect of an intervention to prevent recidivism is likely to be greatest when it is aimed at dynamic risk factors most strongly related to recidivism. This principle, also known as the *needs principle*, has emerged from a series of meta-analyses as an important principle of effective intervention [14–19]. The needs principle also appeared to be important in the treatment of sexual offenders [20]. The dynamic risk/protective factors for both sexual and general offending of female adolescent sexual offenders are still unknown [8]. In recent years, knowledge increased about dynamic *risk* factors that are related to general delinquent behavior in female adolescent *nonsexual* offenders. Various studies have shown that there are both risk factors that are unique for females as well as risk factors shared by males and females (e.g., [21]). Examples of shared risk factors are antisocial peers or attitudes, history of antisocial behaviour, poor parent-child relations, educational difficulties and temperament problems (e.g., [22–25]). Sex differences in risk factors are mainly found for individual and family factors [21]. Examples of risk factors that are specific for females are being a victim of physical and/or sexual abuse, mental health problems, substance abuse, and family problems (e.g., [26–29]).

Far less is known about *protective* factors for delinquency, both for female and male adolescent (sexual and nonsexual) offenders. First, there is a discussion in literature about the definition of protective factors [30, 31]. Some researchers define protective factors as factors that buffer against risks for delinquency [32–35], whereas other researchers assume that protective factors have a direct effect on reducing problem behavior, even where there are no risks present [36]. In recent studies, only support was found for direct effects of protective factors on recidivism and not for indirect effects [11, 37, 38]. Second, there is discussion about the question whether factors are unipolar or bipolar, that is, whether risk factors and protective factors really are two different groups of factors or the same factors, with a risk effect at one extreme and a protective effect at the other. It has been shown in various studies that protective and risk effects often co-occur in the same variables (e.g., [37–39], including variables such as school motivation, parental supervision, relationship with parents, parental stress, and academic performance. In the present study, we focused upon bipolar variables.

In order to be able to refer female juvenile sexual offenders for the appropriate interventions, it is important to know whether and, if so, to what extent there are differences between female sexual and nonsexual offenders in the impact of the risk and protective factors on general recidivism. If the factors associated with general recidivism are the same in both female sexual and nonsexual offenders, then treatment programs developed for mainstream female offenders may also be useful for female sexual offenders in reducing general recidivism. Because there are no previous studies in which FSOs and FNSOs are compared regarding the *impact* of risk and/or protective factors on general recidivism, the aim of the present study was to examine whether there are differences between female adolescent sexual and nonsexual offenders in the impact of dynamic risk and protective factors on general recidivism.

The present study was a follow-up of an earlier study in which we compared female adolescent sexual offenders with both male adolescent sexual offenders and female adolescent nonsexual violent offenders on the prevalence of risk factors [40]. The results of this study showed that female and male adolescent sexual offenders were remarkably similar, whereas female sexual and violent offenders were remarkably different on the measured variables. The present study added to this investigation by (a) examining both risk and *protective* factors, and (b) examining both prevalence and *impact* of the factors.

There was an overlap in the data used in the previous study and the data used in the present follow-up study. In both studies, data was gathered by using the Washington State Juvenile Court Assessment (WSJCA), a risk and needs assessment instrument which comprises two parts: a full assessment and a prescreen [41, 42]. The sample of the earlier study consisted of female adolescents for whom the WSJCA *prescreen* was completed. The sample of the present study was a subset of that sample and consisted of female adolescents who were assessed as having moderate to high risk on the prescreen and for whom the WSJCA *full screen* was completed. The full screen maps out the most important risk and protective factors on a large number of domains whereas the prescreen is a shortened version of the full assessment that aims to provide a quick indication of whether a youth is at low, moderate, or high risk for reoffending.

The aims of the present study were to examine (a) differences between FSOs and FNSOs in background characteristics and the level of general recidivism, (b) differences between FSOs and FNSOs in the prevalence and impact of both risk and protective factors, and (c) the unique contribution of the risk/protective domains to the prediction of recidivism in both FSOs and FNSOs.

## Method

### Sample

For this study, secondary data from the WSJCA validation study were used [41]. The dataset consisted of Washington State probationers with ages 12 to 18 years (*N* = 13,613).The sample of the present study included female adolescents for whom the WSJCA full assessment was performed, which indicates that the participants had a medium to high recidivism risk on the WSJCA pre-screen. From this dataset, the following two groups were selected:

- *Female adolescent sexual offenders (FSOs)*: all female adolescents who committed a felony sexual offense (*n* = 31). Both sexual offenses against a younger child (*n* = 19) and sexual offenses with a peer victim (*n* = 12) were included. Felony sexual offenses include the following offenses: assault to rape, incest and indecent liberties.
- *Female adolescent nonsexual violent offenders (FNSOs)*: all female adolescents who committed a felony violent nonsexual offense (*n* = 407).

### Measures and procedure

Washington State Juvenile Court Assessment (WSJCA).

The WSJCA is a screening and risk assessment instrument developed in Washington State [41, 42]. The WSJCA maps out the most important risk and protective factors for criminal recidivism on a large number of domains. The development of the instrument was based on a review of the following types of research: recidivism prediction literature and validity studies of risk assessment instruments, for example: the Wisconsin Risk Scale [43] and the Youth Level of Service-Case Management Inventory [44], risk and protective factor research, resiliency research and research on effective juvenile delinquency treatment programs (see [41]). The selection of domains and items took place on the basis of this review and was subsequently modified, based on feedback from an international panel of experts [41].

Probation officers perform the full assessments on the basis of information from a structured motivational interview with the youth and youth’s family. Probation officers are trained in conducting the assessment. This training includes reviewing video-taped interviews and the resulting assessment to ensure that the probation officer has mastered the assessment skills. There is a manual available for the full assessment, and quality assurance is an important part of the assessment structure and organization in Washington State [41].

The full assessment measures both static (historical) and dynamic (current) risk and protective factors. In the present study, only dynamic factors were examined, because these factors are used to guide the rehabilitative effort. The dynamic factors are measured over the past six months. The full assessment contains dynamic risk and protective factors in the following domains: school, employment, use of free time, relationships, family, alcohol and drugs, attitude, aggression and skills. The employment domain was excluded from the analysis, because of the large number of missing values (only 9 % are employed).

Most items were rated at a 3-point scale (*strong protective side, neutral middle part, strong risk side*) or a 4-point scale (*strong protective side, weak protective side, weak risk side and strong risk side*). To be able to examine the prevalence and impact separately for risk and protective factors, each item was recoded into two separate items as follows: a protective item (“2” if the strong protective side was present, “1” if the weak protective side was present and “0” if the protective side was absent) and a risk item (“2” if the strong risk side was present, “1” if the weak risk side was present and “0” if the risk side was absent. For example, the response categories of the 4-point item dealing with emotions (*lack skills in dealing with emotions, rarely uses skills in dealing with emotions, sometimes uses skills in dealing with emotions, or often uses skills in dealing with emotions*) were recoded into two separate items as follows: a protective item (“2” *often uses skills in dealing with emotions*, “1” *sometimes uses skills in dealing with emotions* and “0” *lack skills/rarely uses skills in dealing with emotions*), and a risk item ( “2” *lack skills in dealing with emotions,* “1” *rarely uses skills in dealing with emotions*, and “0” *often/sometimes uses skills in dealing with emotions*). Another example, the response categories of the 3-point item pro-social community ties (*no prosocial community ties, some pro-social community ties, strong pro-social community ties*) were recoded into two separate items as follows: a protective item (“2” *strong pro-social community ties* and “0” *no social community ties or some pro-social community ties*) and a risk item (“2” *no prosocial community ties* and “0” *strong/some pro-social community ties*).

The impact of the risk and protective factors was examined separately because there may be a difference in the strength of the risk effect and the strength of the protective effect of a bipolar variable [39]. For example: “does not believe education of value” might be a relative strong predictor of recidivism, whereas “believes getting education of value” might offer only a small protection against recidivism.

For each domain, a total risk score was calculated by adding the scores of the individual risk factors within that domain and a total protective score was calculated for each domain by adding the scores of individual protective factors within the domain (risk/protective domain scales). So within scales, items were weighted equally, regardless of the number of scale points of the items (e.g., 0-1 vs. 0-2, etc.). Then, Cronbach’s alphas were calculated for each domain scale. Items were deleted from a domain scale in case that improved Cronbach alpha. Risk/protective domain scales with alphas lower than .70 were excluded from further analysis. Cronbach’s alphas for the remaining risk and protective domain scales are presented in Table 1. The protective and risk items that are part of different domain scales are presented in Table 2.

**Table 1:** Cronbach’s alphas for the risk and protective domain scores (*N* = 438)

	Risk scores				Protective scores			
	M	SD	N of items	α	M	SD	N of items	α
School	6.44	4.75	8	.84	2.87	2.82	8	.81
Relationships	3.29	1.93	4	.70	3.43	2.10	5	.70
Family	7.78	4.98	11	.83	6.07	3.47	11	.83
Alcohol/drugs	.73	.85	2	.74	1.31	.84	2	.72
Attitude	6.39	4.83	9	.86	6.62	4.79	9	.83
Aggression	5.15	3.00	5	.78	2.84	2.83	5	.74
Skills	5.50	4.99	10	.87	8.10	4.89	10	.91

**Table 2:** Risk and protective items that are part of the different domain scales

Risk domain	Risk end	Scale points	Protective end	Scale points
School	Behavior problems	0-3	Good behavior at school	0-2
	Poor academic performance	0-2	Good academic performance	0-3
	Truancy	0-3	Good attendance	0-2
	Suspended or Dropped out or Expelled	0-3	Close relationship with teachers Participation in school activities	0-2
	Not interested or involved in school activities	0-2	Believes school is encouraging	0-2
	Youth does not believe school is encouraging	0-2	Believes getting education of value	0-1
	Youth does not believe education of value	0-2	Likely to graduate	0-1
	Not likely to graduate	0-2		0-1
Relationships	Antisocial friends or gang membership	0-3	Only pro-social friends	0-1
	Admires or emulates antisocial peers,	0-2	No admiration of anti-social peers	0-1
	Rarely resists antisocial peer influence	0-2	Resist antisocial peer influence	0-2
	Romantically involved with an antisocial person	0-1	Positive adult non-family relationships	0-3
			Prosocial community ties	0-2
Family	Low family income	0-2	High annual income	0-2
	Poor relationship with parents	0-1	Close relationship with father / mother	0-1
	(Serious) conflicts in the family	0-3	Few and/or well managed conflicts	0-1
	Inadequate parental supervision	0-2	Consistent, good parental supervision	0-1
	Youth (consistently) disobeys family	0-2	Usually obeys and following family rules	0-1
	No family support network	0-1	Strong family support network	0-2
	Poor parental punishment	0-2	Consistent, appropriate parental punishment	0-1
	Poor parental reward	0-2	Consistent, appropriate parental reward	0-1
	Family little or not willing to support youth	0-3	Family willing to support youth	0-1
	Family provides no opportunities to participate in family activities/decisions	0-2	Family provides opportunities to participate in family activities/decisions	0-1
	Youth is currently kicked out of home or is a runaway	0-2	Youth has not run away/kicked out of home	0-1
Alcohol/drugs	Current alcohol abuse	0-2	No current alcohol use	0-1
	Current drug abuse	0-2	No current drug use.	0-1
Attitude	Impulsiveness (usually acts before thinking)	0-2	Uses self control (thinks before acting)	0-2
	No or little control over antisocial behavior	0-2	Belief in control over anti-social behavior	0-2
	No or little empathy, remorse, or sympathy	0-2	Empathy, remorse, or sympathy for victims	0-2
	No or little respect for other’s property	0-3	Respect for others’ property,	0-2
	No or little respect for authority figures	0-3	Respect for authority figures,	0-2
	No or little respect for rules/social conventions	0-3	Respect for rules/social conventions	0-2
	Does not accept responsibility for behavior	0-3	Accepts responsibility for behavior	0-2
	Does not think he/she can comply with measures	0-2	Thinks they can comply with measures	0-1
	Low aspirations for better life (little sense of purpose or plans for better life)	0-2	High aspirations for better life	0-2
Aggress	Low frustration tolerance	0-2	Tolerance for frustration	0-2
	Believes verbal aggression is sometimes or often appropriate to solve a conflict	0-2	Believes verbal aggression to solve a conflict is rarely or never appropriate	0-2
	Believes physical aggression is sometimes or often appropriate to solve a conflict	0-2	Believes physical aggression to solve a conflict is rarely or never appropriate	0-2
	Lacks alternatives to aggression	0-2	(Often) uses alternatives to aggression	0-3
	Hostile interpretation of other’s behavior/intentions	0-2	Primarily positive interpretation of other’s behavior/intentions	0-2
Skills	Poor consequential thinking	0-1	Good consequential thinking	0-3
	Does not set any goals/set unrealistic goals	0-2	Set realistic goals	0-2
	Poor problem-solving behavior	0-1	Applies appropriate solutions	0-3
	Lacks basic social skills	0-1	Uses advanced social skills	0-3
	Lacks skills in dealing with difficult situations	0-2	Uses skills in dealing with difficult situations	0-2
	Lack of skills in dealing with feelings/emotions	0-2	Uses skills in dealing with emotions	0-2
	Problems in controlling internal triggers	0-2	Actively monitors and controls internal triggers	0-2
	Problems in controlling external triggers	0-2	Actively monitors and controls external triggers	0-2
	Lacks techniques to control impulsive behavior	0-2	Uses techniques to control impulsive behavior	0-3
	Cannot analyze the situation for use of a prosocial skill	0-2	Can select the best time and place to use the best pro-social skill	0-3

### Analyses

First, analysis of variance (ANOVA) was used to determine whether there were differences in the prevalence of risk and protective factors for general recidivism between FSOs and FNSOs. Because of multiple testing (16 tests), *p*-values were adjusted using the method of Benjamini and Hochberg [45] to control the false discovery rate.

Second, associations between risk/protective factors and recidivism were examined in both FSOs and FNSOs. Point-biserial correlation coefficients (*r*_*pb*_) were used because one of the variables was dichotomous (recidivism) and one of variables was interval (the sum of risk/protective factors). To control for the false discovery rate of multiple testing (28 tests), *p*-values were adjusted using the method of Benjamini and Hochberg [45].

Fisher’s z tests were calculated to assess the significance of the differences between the correlations of the two offender groups. Again, adjusted *p*-values were calculated using the method of Benjamini and Hochberg [45] to control for the false discovery rate of multiple testing (14 tests).

Finally, multivariate logistic regression analyses were performed to examine the unique contribution of the risk/protective domain scores to the prediction of recidivism in both FSOs and FNSOs.

### Ethical approval

Formal Institutional Review Board (IRB) approval to conduct this study was not required, as this study involved secondary data analysis on de-identified data, which does not pose harm to the subjects and therefore does not necessitate IRB regulation. Accordingly, this study was ethically conducted based on the rules maintained by the Faculty Ethics Review Board (FMG-UvA) of the University of Amsterdam, The Netherlands. The Washington State Institute for Public Policy has given permission to use the data for this study.

## Results

The background characteristics and recidivism rates for FSOs and FNSOs are presented in Table 3. In comparison with FNSOs, European Americans were overrepresented and African Americans were underrepresented in the FSO group. There were no significant differences in average age and recidivism rates between FSOs and FNSOs.

**Table 3:** Background Characteristics and recidivism rates for the female adolescent sexual and nonsexual offenders

	FSOs	FNSOs	*F*
	(*n* = 31)	(*n* = 407)	
Ethnicity:			
European Americans	80.7 %	48.7 %	12.07**
African Americans	3.2 %	17.0 %	4.06*
Hispanic Americans	6.5 %	8.4 %	.14
Other	3.2 %	9.1 %	1.25
Unknown	6.5 %	17.0 %	2.34
Average age:			
At the time of the assessment	15.10 (*SD* = 1.56)	15.40 (*SD* = 1.34)	1.46
At first offense	13.42 (*SD* = 1.79)	13.58 (*SD* = 1.60)	.30
Recidivism rates:			
Total Recidivism	23 %	36 %	2.24
Felony Recidivism	10 %	18 %	1.37
Violent Felony Recidivism	3 %	6 %	.38

*p < .05, **p < .01

The prevalence of dynamic risk and protective factors in FSOs and FNSOs are presented in Table 4. In FSOs, fewer dynamic risk factors were present in the domains of school, relationships, family, attitude (trend-significant), and aggression than in FNSOs. In addition, in FSOs more dynamic protective factors were present in the domains of school, relationships, family, attitude, aggression, and skills (trend-significant) than in FNSOs. The total risk score is about 1.5 times larger in FNSOs (39.56) than in FSOs (25.52), whereas the total protective score is about 1.4 times larger in FSOs (50.71) than in FNSOs (36.22).

**Table 4:** Prevalence of dynamic risk and protective factors in female adolescent sexual and nonsexual offenders

	Dynamic risk factors			Dynamic protective factors		
	FSOs	FNSOs	*F*	FSOs	FNSOs	*F*
	(*n* = 31)	(*n* = 407)		(*n* = 31)	(*n* = 407)	
School	4.39	6.90	7.16^a^	7.00	4.69	11.54^a^
Relationships	1.94	3.40	17.13^a^	4.77	3.33	14.05^a^
Family	5.45	10.00	16.70^a^	13.58	9.67	22.73^a^
Alcohol/drugs	.68	1.01	2.03	1.52	1.30	1.97
Attitude	5.55	7.36	3.93^b^	8.74	6.62	5.44^a^
Aggression	3.10	5.30	16.07^a^	5.39	2.64	28.69^a^
Skills	4.42	5.58	1.56	9.71	7.98	3.67^b^
Total score	25.52	39.56	16.86^a^	50.71	36.22	19.77^a^

^a^Significant after controlling for the false discovery rate using the method of Benjamini and Hochberg (1995), using a .05 level for the false discovery rate

^b^trend-significant after controlling for the false discovery rate using the method of Benjamini and Hochberg (1995), using a .10 level for the false discovery rate

Table 5 shows the point-biserial correlations (*r*_*pb*_) between the dynamic risk factors and recidivism and between the dynamic protective factors and recidivism separately for FSOs and FNSOs. The base rates (recidivism rates) differed between FSOs and FNSOs; therefore, values for small, medium, and large effect sizes for point-biserial correlations (*r*_pb_) were calculated for the different base rates in FSOs and FNSOs, based on a conversion formulae (after Rosental, 1991; Swets, 1986) provided by Rice and Harris [46]. For a 23 % base rate (recidivism in FSOs), the *r*_pb_ values for small, medium, and large are .084, .206, and .319 respectively and for a 36 % base rate (recidivism in FNSOs) the *r*_pb_ values for small, medium, and large are .096, .233, and .358 respectively.

**Table 5:** Correlations between the dynamic risk/protective factors and recidivism, separately for female adolescent sexual and nonsexual offenders

	Dynamic risk factors			Dynamic protective factors		
	FSOs	FNSOs	*Z*	FSOs	FNSOs	*Z*
	(*n* = 31)	(*n* = 407)		(*n* = 31)	(*n* = 407)	
School	.25	.01	1.26	-.26	-.19^a^	.38
Relationships	.30	.16^a^	.76	-.29	-.13^a^	.86
Family	.48^a^	.12^a^	2.06^b^	-.46^a^	-.17^a^	1.66
Alcohol/drugs	.09	.04	.26	-.16	-.08	.42
Attitude	.30	.26^a^	.22	-.36^b^	-.27^a^	.51
Aggression	.53^a^	.20^a^	1.98^b^	-.39^b^	-.23^a^	.91
Skills	.29	.20^a^	.49	-.30	-.18^a^	.65

^*^Significant after controlling for the false discovery rate using the method of Benjamini and Hochberg (1995), using a .05 level for the false discovery rate

^+^Trend-significant after controlling for the false discovery rate using the method of Benjamini and Hochberg (1995), using a .10 level for the false discovery rate

*z* = Fisher’s z significance test for the difference between groups in the strength of the correlations between the risk factors and recidivism

In addition, due to the differences in sample sizes, the strength of a correlation had to be relatively large to be considered significant in FSOs compared to FNSOs. In FSOs, risk factors in domains of family and aggression were significantly related to recidivism and protective factors in the domains of family, attitude (trend-significant), and aggression (trend-significant). All the above mentioned effects are large effects (*r*_pb_ > .319).

In FNSOs, risk factors the domains of relationships, family, attitude, aggression, and skills were significantly related to recidivism and protective factors in the school, relationships, family, attitude, aggression and skills domain. Most of these significant correlations corresponded with small effects (*r*_*pb*_ < .233) with the exception of risk and protective factors in the attitude domain (medium effects).

Fisher’s z tests were calculated to assess the significance of the differences between the correlations of the two offender groups. The relation between the dynamic risk factors and recidivism was stronger in FSOs than in FNSOs for the family and aggression domains (trend-significant).

Finally, to examine the unique contribution of the risk and protective factors to the prediction of recidivism, multivariate logistic regression analyses were performed separately for FSOs and FNSOs and separately for the risk and protective factors (see Table 6 and Table 7). In FSOs, risk factors in the aggression (odds ratio = 1.80, *p* < .05) domains and protective factors in the family domain (odds ratio = .60, *p* < .05) were uniquely related to recidivism. In FNSOs, risk factors in the attitude domain (odds ration = 1.12, *p* < .001) and protective factors in the attitude domain (odds ratio = .89, *p* < .001) were uniquely related to recidivism.

**Table 6:** Logistic regression coefficients predicting recidivism from risk factors for female adolescent sexual and nonsexual offenders (method: Forward Wald)

	FSOs (*n* = 31)				FNSOs (*n* = 407)			
	B	SE	Wald	Exp(B)	B	SE	Wald	Exp(B)
Aggression	.59	.24	5.93*	1.80				
Attitudes					.11	.02	25.38***	1.12
Constant	−3.61	1.26	8.22**	.03	−1.43	.20	49.36***	.24
χ(df)	9.68(1)**				27.25(1)***			

*p < .05, **p < .01, ***p < .001

**Table 7:** Logistic regression coefficients predicting recidivism from protective factors for female adolescent sexual and nonsexual offenders (method: Forward Wald)

	FSOs (*n* = 31)				FNSOs (*n* = 407)			
	B	SE	Wald	Exp(B)	B	SE	Wald	Exp(B)
Family	-.50	.22	5.09*	.60				
Attitudes					-.12	.03	25.05***	.89
Constant	.66	.84	.62	1.93	.13	.18	.52	1.14
χ(df)	6.93(1)**				28.01(1)***			

*p < .05, **p < .01, ***p < .001

## Discussion

This study examined differences in the prevalence and impact of dynamic risk and protective factors on general recidivism between female adolescent sexual and nonsexual offenders. Results showed that in the sexual offending group, considerably more protective and fewer risk factors were present in the domains of school, relationships, family, attitude and aggression, than in the nonsexual offenders. The total risk score was about 1.5 times larger in the nonsexual offending group than in the sexual offending group, whereas the opposite was true for the total protective score, which was about 1.4 times larger in sexual offenders than in nonsexual offenders. The only previous study comparing female adolescent sexual and nonsexual offenders with regard to the prevalence of risk factors yielded the same pattern of results, with the sexual offenders scoring lower than the nonsexual offenders on criminal history, school behavior problems and fighting [47]. In addition, the findings regarding the prevalence of risk factors are consistent with findings from available comparisons of *male* adolescent sexual and nonsexual offenders. Seto and Lalumiere (2010) found in their meta-analysis [48] that risk factors for general delinquency (criminal histories, antisocial peers, and substance use) were less common among sexual offenders than among nonsexual offenders.

The correlations between risk/protective factors and recidivism were higher in female adolescent sexual offenders than among female adolescent nonsexual offenders in all domains, with the differences being trend-significant in the domains of family and aggression. The potential effect on general recidivism of interventions that address factors in these domains is therefore also expected to be relatively large in female adolescent sexual offenders. There are no previous studies in which a comparison is made between female sexual and nonsexual offenders in the *strength* of the relation between risk/protective factors and general recidivism. Research of this kind is only possible where the samples are sufficiently large, which is rarely the case in research on female sexual offenders. Recently, we examined differences between *male* adolescent sexual and nonsexual offenders in the impact of risk factors on general recidivism and we also found the impact to be greater in the sexual offending group [49].

The results of the present study showed which risk/protective factors were uniquely related to general recidivism for female adolescent sexual and nonsexual offenders. In female adolescent sexual offenders, the family and aggression domains seem to be particularly important, given that risk and/or protective factors in these domains uniquely contributed to recidivism in these youths. Thus, addressing risk/protective factors pertaining to the family and aggression domains may be effective in interventions aimed at reducing criminal recidivism among female adolescent sexual offenders. Examples of these factors are improving relationship with parents, improving parental supervision, strengthening family supportive network, enhancing conflict resolution skills and improving frustration tolerance. Family Functional Therapy (FFT; [50, 51] is an example of an intervention that addresses family risk factors. FFT includes behavioural contracting, communication skills, specification of rules, and a token reinforcement system, as techniques to improve family functioning and reduce delinquent behavior. An example of an intervention that addresses risk factors in the aggression domain is Aggression Regulation Therapy (ART; [52]), a group training in which three different skills are taught: anger management, social skills and moral reasoning.

In female adolescent nonsexual offenders, risk and protective factors in the attitude domain were uniquely related to recidivism and, therefore, addressing these factors may be effective in interventions aimed at reducing criminal recidivism. Examples of these factors are acceptance of responsibility for behavior, respect for rules, social conventions and/or authority figures and improving aspirations for better life. Various review studies have shown that cognitive-behavioral therapy is an effective intervention targeting criminogenic attitudes and subsequent delinquent behavior [12, 53].

Some limitations of this study need to be mentioned. First, the sample size for female adolescent sexual offenders was still relatively small (*n* = 31), despite the extensive dataset that was available. The statistical power to detect a medium-sized effect (two tailed) with a sample of *n* = 31 is 39 %, and the statistical power to detect a large effect is 87 %. In general, a statistical power to detect a medium-sized effect of 80 % is considered adequate [54]. Second, the sample of this study consisted of female adolescent sexual and nonsexual violent offenders on probation with an estimated moderate to high criminal offense recidivism risk. Therefore, the results of the study cannot be generalized to the total population of female adolescent sexual and nonsexual violent offenders. Third, the WSCJA was not designed to provide an in-depth examination of risk factors. Instead, it is a risk assessment tool that is designed to be used by juvenile justice professionals and clinicians to summarize juveniles’ risks and (criminogenic) needs, classify their overall risk level, and plan treatment and supervision strategies. Fourth, there are no research results available of the interrater reliability of the WSJCA. However, quality assurance is an important part of the assessment structure and organization in Washington State, and probation officers receive intensive training to adequately administer and reliably score the WSJCA (Barnoski, 2004a). Fifth, the risk/protective domain scales were constructed by adding the scores of individual risk/protective risk factors. So, within scales, items were weighted equally, regardless of the number of scale points of the items. We have chosen to weight items equally (regardless of point ranges of items) instead of using unequal weights, for example calculated by factor analysis, because computed weights usually will vary from sample to sample and we examined two different samples (FSOs and FNSOs). Because the number of scale points varied per item, we have implicitly chosen to give items with larger scale ranges more weight than items with smaller scale ranges. This might have affected the results of the study. Sixth, some of the created total risk/protective scales consisted of only few items (e.g., the risk and protective scales of the alcohol/drugs domain and the risk and protective scales of the relationship domain). As a result, the distributions of those variables were skewed and parametric statistical assumptions of calculating ANOVAs, correlations and regression coefficients were violated. This might have affected the results of the study. However, there have been a number of studies that have shown that the performed statistics (ANOVA, Pearson correlations and regression) are robust for skewed and non-normal distributions (Norman, 2010). Finally, the follow-up period of eighteen months was too short to adequately measure sexual recidivism because of the low incidence of sexual offenses in female adolescent offenders. The study would have provided more useful information if it also had included associations with sexual recidivism.

## Conclusions

The results of this study add to the scarce literature on female adolescent sexual offenders by being the first to study the strength of the relationships between risk/protective factors and general recidivism. The results of this study have important implications for clinical practice. First, this study showed that dynamic risk and/or protective factors in all domains were more strongly related to general recidivism in female adolescent sexual offenders than in the nonsexual offenders, with the differences being significant in de domains of family and aggression. The potential effect on general recidivism of interventions that address these factors is therefore also expected to be relatively large in the sexual offending group. These results indicates that treatment programs developed for mainstream female offenders may also be useful for female sexual offenders in reducing general recidivism. Moreover, the potential effect of these treatments may even be greater for sexual offenders than for nonsexual offenders because of the relatively high impact of the risk factors on general recidivism. Second, the results of multivariate analyses showed that for the sexual offending group, risk/protective factors in the family and aggression domains are especially important, whereas for the nonsexual offending group, risk/protective factors in the attitude domain are especially important. These results are of importance for determining the main focus of treatment. Finally, the results of this study contribute to the limited knowledge about protective factors. It was shown that not only risk but also protective factors were relatively important for female adolescent sexual offenders, as they were more strongly related to general recidivism in female adolescent sexual offenders than in the nonsexual offenders. These results suggest that clinicians should address both risk and protective factors in order to prevent recidivism in female adolescent sexual offenders. In future research, the impact of risk and protective factors on *sexual* recidivism should be examined in female adolescent sexual offenders. Until now, it is unknown whether there are specific risk and protective factors for sexual offending in female adolescents, while this knowledge is very useful to inform treatment decisions and develop treatment programs that fit the needs of female adolescents who offend sexually.

## References

[1] Becker JV (1990). Treating adolescent sex offenders. Prof Psychol Res Pr.

[2] Becker JV (1998). What we know about the characteristics and treatment of adolescents who have committed sexual offenses. Child Maltreat.

[3] Worling JR, Långström N, Barbaree HE, WL M s (2006). Risk of sexual recidivism in adolescents who offend sexually: Correlates and assessment. The Juvenile Sex Offender.

[4] Gottfredson MR, Hirschi T (1990). A General Theory of Crime.

[5] France KG, Hudson SM, Barbaree HE, Marshall WL, Hudson SM (1993). The conduct disorders and the juvenile sex offender. The Juvenile Sex Offender.

[6] Cortoni F, Hanson RK, Coache M (2010). The recidivism rates of female sexual offenders are low: a meta-analysis. Sex Abuse-J Res Tr.

[7] McCann K, Lussier P (2008). Antisociality, sexual deviance, and sexual reoffending in juvenile sex offenders. A meta-analytical investigation. Youth Violence Juv Justice.

[8] Gannon TA, Cortoni F (Eds.): Female sexual offenders: Theory, assessment and treatment. Chichester, UK: Wiley-Blackwell. Com, 2010.

[9] Howell JC (2003). Diffusing research into practice using the comprehensive strategy for serious, violent, and chronic juvenile offenders. Youth Violence Juv Justice.

[10] Loeber R, Farrington DP, Stouthamer-Loeber M, White HR (2008). Violence and Serious Theft: Development and Prediction from Childhood to Adulthood.

[11] Stouthamer-Loeber M, Loeber R, Wei E, Farrington DP, Wikström P-OH (2002). Risk and promotive effects in the explanation of persistent serious delinquency in boys. J Consult Clin Psychol.

[12] Andrews DA, Bonta J (2010). The Psychology of Criminal Conduct.

[13] Loeber R, Slot W, Stouthamer-Loeber M, Loeber R, Koot HM, Slot NW, Van der Laan PH, Hoeve M (2008). A cumulative developmental model of risk and promotive factors. Tomorrow’s criminals: The development of child delinquency and effective interventions.

[14] Andrews DA, Bonta J (2010). Rehabilitating criminal justice policy and practice. Psychol Pub Pol'y L.

[15] Andrews DA, Zinger I, Hoge RD, Bonta J, Gendreau P, Cullen FT (1990). Does correctional treatment work? A clinically relevant and psychologically informed meta-analysis. Criminol.

[16] Dowden C, Andrews DA (1999). What works for female offenders: a meta-analytic review. Crime Delinq.

[17] Dowden C, Andrews DA (2000). Effective correctional treatment and violent reoffending: a meta-analysis. Can J Criminol.

[18] Gendreau P, Harland AT (1996). The Principles of Effective Intervention with Offenders. Choosing Correctional Interventions that Work: Defining the Demand and Evaluating the Supply.

[19] Gendreau P, Smith P, French SA, Cullen FT, Wright JP, Blevins KR (2006). The theory of effective correctional intervention: Empirical status and future directions. Taking stock: The status of criminological theory: Vol. 15. Advances in criminological theory.

[20] Hanson RK, Bourgon G, Helmus J, Hodgson S (2009). The principles of effective correctional treatment also apply to sexual offenders: A meta-analysis. Crim Justice Behav.

[21] Wong TM, Slotboom AM, Bijleveld CC (2010). Risk factors for delinquency in adolescent and young adult females: a European review. Eur. J. Criminol.

[22] Hubbard DJ, Pratt TC (2002). A meta-analysis of the predictors of delinquency among girls. J Offender Rehabil.

[23] Lipsey MW, Cook TD (1992). Juvenile Delinquency Treatment. A Meta-Analytic Inquiry into the Variability of Effects. Meta-Analysis for Explanation.

[24] Moffitt TE (1993). Adolescence-limited and life-course-persistent antisocial behavior: a developmental taxonomy. Psychol Rev.

[25] Simourd L, Andrews DA (1994). Correlates of delinquency: a look at gender differences. Forum on Corrections Research.

[26] Hubbard DJ, Pratt TC (2002). A meta-analysis of the predictors of delinquency among girls. J Offender Rehabil.

[27] Salisbury EJ, Van Voorhis P, Spiropoulos GV (2009). The predictive validity of a gender-responsive needs assessment: an exploratory study. Crime Delinq.

[28] Van der Put CE, Deković M, Hoeve M, Stams GJJM, Van der Laan PH, Langewouters F (2014). Risk assessment of girls: are there any sex differences in risk factors for re-offending and in risk profiles?. Crime Delinq.

[29] Zahn M (2009). The Delinquent Girl.

[30] Dekovic M (1999). Risk and protective factors in the development of problem behavior during adolescence. J Youth Adolesc.

[31] Luthar SS, Cicchetti D, Becker B (2000). The construct of resilience: a critical evaluation and guidelines for future work. Child Dev.

[32] Fergusson DM, Lynskey MT (1996). Adolescent resiliency to family adversity. J Child Psychol Psychiatry.

[33] Pollard JA, Hawkins JD, Arthur MW (1999). Risk and protection: are both necessary to understand diverse behavioral outcomes in adolescence?. Soc Work Res.

[34] Rutter M (1987). Psychosocial resilience and protective mechanisms. Am. J. Orthopsychiatry.

[35] Rutter M, Lahey BB, Moffitt TE, Caspi A (2003). Crucial Paths from Risk Indicator to Causal Mechanism. Causes of Conduct Disorder and Juvenile Delinquency.

[36] Sameroff AJ, Bartko WT, Baldwin A, Baldwin C, Seifer R, Lewis M, Feiring C (1998). Family and Social Influences on the Development of Child Competence. Families, Risk, and Competence.

[37] Farrington DP, Loeber R, Jolliffe D, Pardini DA, Loeber R, Farrington DP, Stouthamer-Loeber M, Raskin White H (2008). Promotive and Risk Processes at Different Life Stages. Violence and Serious Theft. Development and Prediction from Childhood to Adulthood.

[38] Van der Laan AM, Veenstra R, Bogaerts S, Verhulst FC, Ormel J (2010). Serious, minor, and non-delinquents in early adolescence: the impact of cumulative risk and promotive factors. The TRAILS study. J Abnorm Child Psych.

[39] Stouthamer-Loeber M, Loeber R, Farrington DP, Zhang Q, Van Kammen WB, Maguin E (1993). The double edge of protective and risk factors for delinquency: interrelations and developmental patterns. Dev Psychopathol.

[40] Van der Put CE, Van Vug ES, Stams GJJM, Hendriks J (2014). Psychosocial and developmental characteristics of female adolescents who have committed sexual offenses. Sex Abuse-J Res Tr.

[41] Barnoski R (2004). Assessing Risk for Re-offense: Validating the Washington State Juvenile Court Assessment.

[42] Barnoski R (2004). Washington State Juvenile Court Assessment Manual, Version 2.1.

[43] Baird SC, Storrs GM, Connelly H (1984). Classification of Juveniles in Corrections: A Model Systems Approach.

[44] Hoge RD, Andrews DA (1994). The Youth Level of Service/Case Management, Inventory and Manual.

[45] Benjamini Y, Hochberg Y. Controlling the false discovery rate: a practical and powerful approach to multiple testing, Journal of the Royal Statistical Society. Series B (Methodological). 1995;57(1):289–300.

[46] Rice ME, Harris GT (2005). Comparing effect sizes in follow-up studies: ROC Area, Cohen's d, and r. Law Hum Behav.

[47] Kubik EK, Hecker JE, Righthand S (2003). Adolescent females who have sexually offended: comparisons with delinquent adolescent female offenders and adolescent males who sexually offend. J Child Sex Abus.

[48] Seto MC, Lalumiere ML (2010). What is so special about male adolescent sexual offending? A review and test of explanations through meta-analysis. Psychol Bull.

[49] Van der Put CE, Van Vugt ES, Stams GJJM, Deković M, Laan V d (2013). Risk profiles of different types of juvenile sex offenders: Differences in the prevalence and impact of risk factors for general recidivism among different types of juvenile sex offenders and non-sex offenders. Sexual Abuse J Res Tr.

[50] Alexander JF, Parson BV (1982). Functional Family Therapy: Principles and Procedures.

[51] Sexton T, Alexander JF (2004). Functional Family Therapy: Clinical Training Manual.

[52] Goldstein AP, Glick B, Gibbs JC (1998). Aggression Replacement Training: A Comprehensive Intervention for Aggressive Youth.

[53] Lipton DS, Pearson FS, Cleland CM, Yee D, McGuire J (2002). The effectiveness of cognitive-behavioral treatment: meta-analytic outcomes from the CDATE project. Offender rehabilitation and treatment/effective programmes and policies to reduce re-offending.

[54] Cohen J (1988). Statistical power analysis for the behavioral sciences.

